# Supplementary material for: Cytoplasmic Colocalization of RXRα and PPARγ as an Independent Negative Prognosticator for Breast Cancer Patients
Source: Cells. 2022 Apr 6;11(7):1244. doi: 10.3390/cells11071244 (PMC8997589; doi:10.3390/cells11071244)
Supplement: Supplementary file 1 [file cells-11-01244-s001.zip › cells-1634388-supplementary.pdf]

Supplementary Table S1

	PPARγ	RXRα
Positive controls	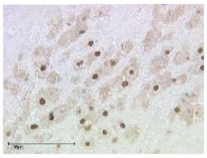 Placenta	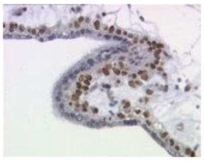 Placenta
Negative controls	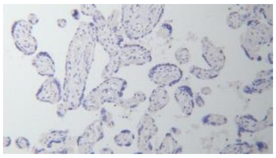 Placenta	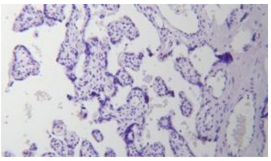 Placenta
materials	**Primary antibody:**<br>Anti-PPAR gamma; Rabbit IgG polyclonal. Conc: 1mg/ml<br>Company: Abcam<br>Order number: ab59256<br><br>**Detection System:**<br>ZytoChem Plus HRP Polymer System (Mouse/Rabbit) (Zytomed; No. POLHRP-100)<br><br>**Chromogen Substrate Solution:**<br>Liquid DAB+ Substrate Chromogen System (Dako; Order No. K3468).	**Primary antibody:**<br>Anti RxR alpha; clone K8508 (mouse IgG2a); Conc: 1 mg/ml.<br>Company: Perseus Proteomics<br>Order number: PP-K8508-10<br><br>**Detection system**:<br>Vectastain Elite mouse -IgG kit (Linaris; No.PK-6102).<br><br>**Chromogen Substrate Solution:**<br>Liquid DAB+ Substrate Chromogen System (Dako; Order No.K3468).
Staining Protocol	1. Deparaffinize in xylene<br>2. Blocking: **20 min in 3% H_2O_2 in methanol.**<br>3. Demask by heat pretreatment in pressure cooker with sodium citrate buffer pH 6.0<br>4. 5 min **Blocking Solution.**<br>5. **primary antibody** 16h overnight at 4°C; dilution: 1:100 in PBS<br>6. **30 min HRP polymer**<br>7. **30 sec. substrate staining with DAB**<br>8. **2 min. counterstaining**<br>9. **bluing for 5 min in tap water**<br>10. cover with "Eukitt"	1. Deparaffinize in xylene<br>2. Blocking: **20 min in 3% H_2O_2 in methanol.**<br>3. Demask by heat pretreatment in pressure cooker with sodium citrate buffer pH 6.0<br>4. **3 min. Power Block (BioGenex; No.HK0851008)**<br>5. **4°C overnight (16h): primary antibody** Dil.: 1:200 in in PBS<br>6. **30 min secondary antibody** = biotinylated Link-Ak, which binds the ABC complex<br>7. **30 min ABC complex**<br>8. **1 min substrate staining with DAB+Chromogen**<br>9. **1 min. counterstaining**<br>10. bluing for 3 min in tap water.<br>11. Covering with "Eukitt"
Staining	nucleus and cytoplasm	nucleus and cytoplasm

Supplementary Figure 1

A: RXR in normal breast tissue

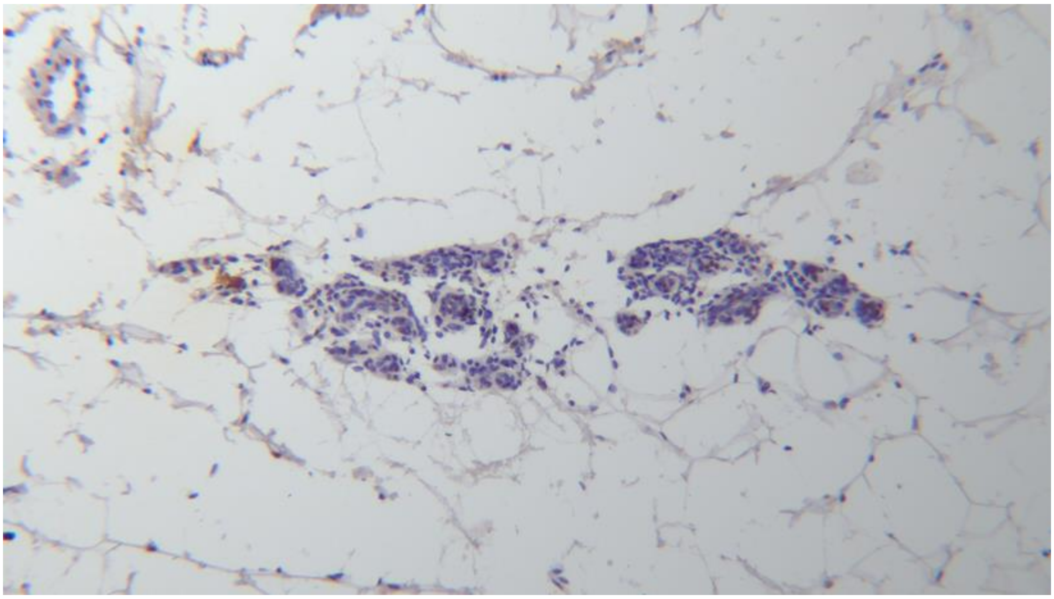

B: PPAR in normal breast tissue

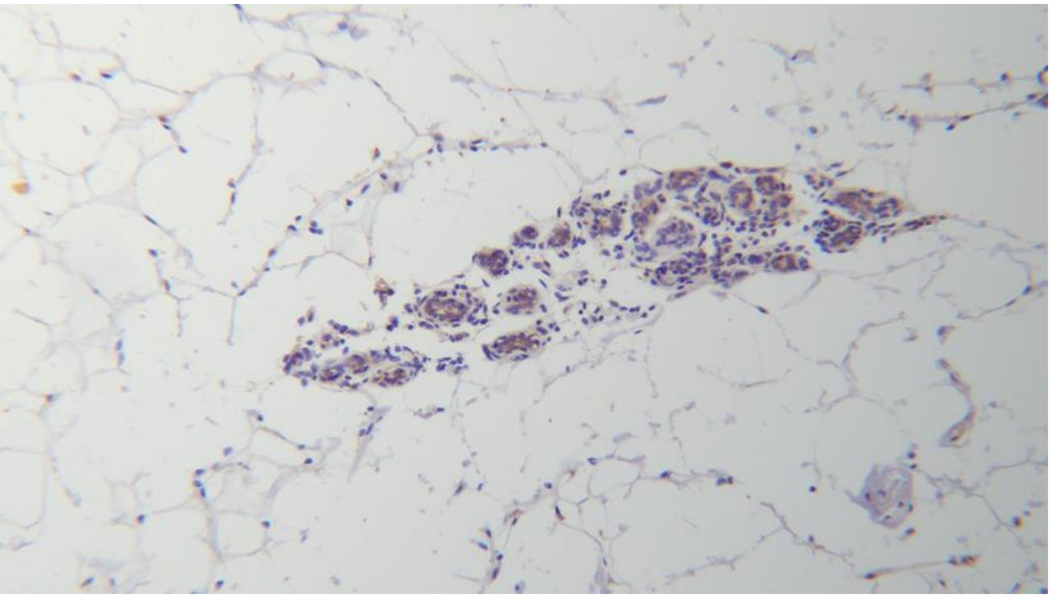

Supplementary figure 2

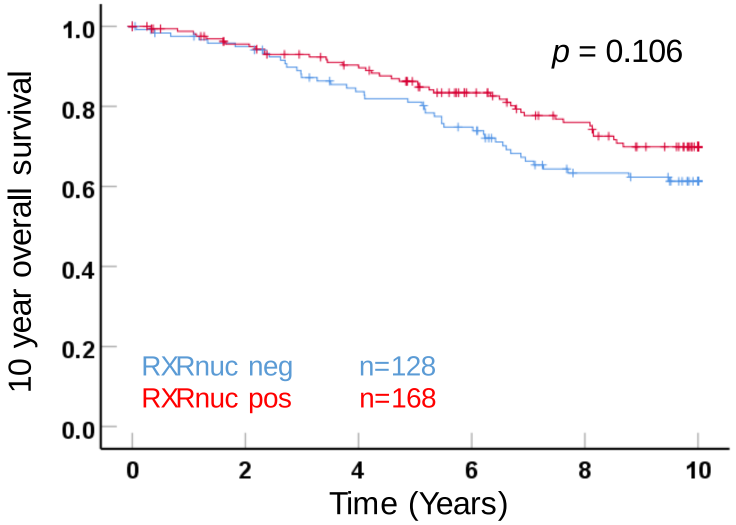

A: Nuclear RXR expression

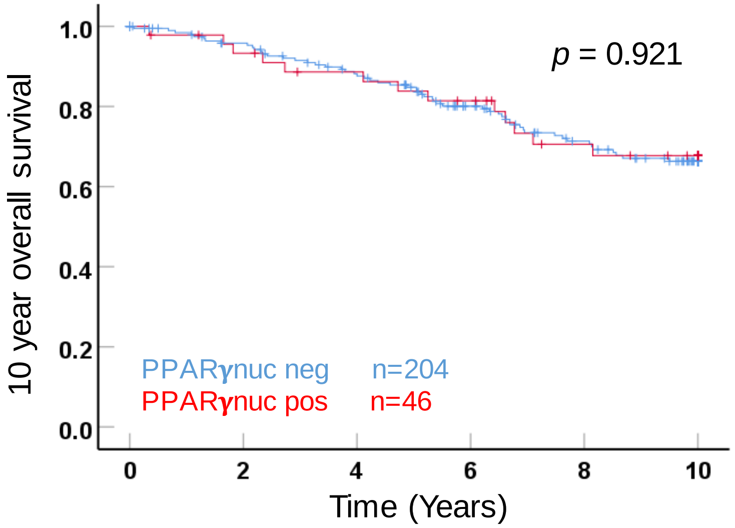

B: Nuclear PPARγ expression

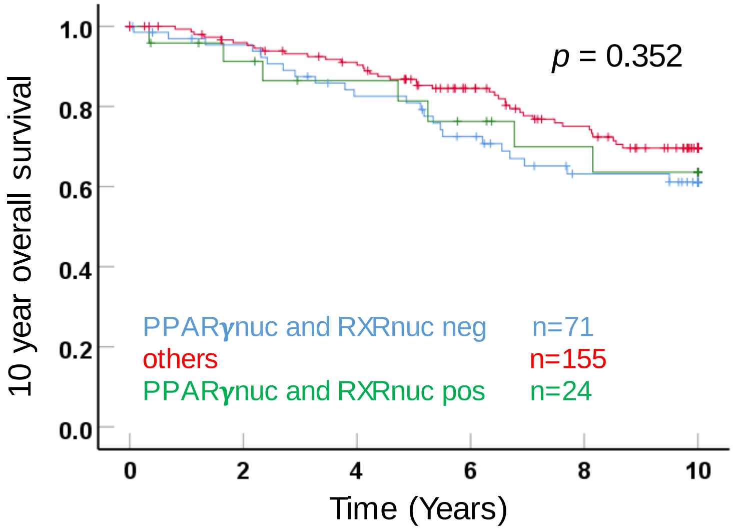

C: Combined Nuclear expression RXR and PPARγ

Supplementary Table S2 (belonging to Figure 2A).

Subgroups	Total number of cases	Estimate of survival time in years
PPARg low/RXR neg	49	8,817
PPARg low/RXR pos.	70	8,603
PPARg high/RXR neg.	46	8,508
PPARg high/RXR pos.	85	7,704
overall	250	8,311

Supplementary Table S3 (belonging to Figure 2B).

Subgroups	Total number of cases	Estimate of survival time in years
Other cases	165	8,64
PPARg high/RXR pos.	85	7,704
overall	250	8,311

Supplementary Table S4 (belonging to Figure 3A).

Subgroups	Total number of cases	Estimate of survival time in years
PPARg low/RXR neg	49	5,299
PPARg low/RXR pos.	70	5,412
PPARg high/RXR neg.	46	5,366
PPARg high/RXR pos.	85	4,169
overall	250	4,943

Supplementary Table S5 (belonging to Figure 3B).

Subgroups	Total number of cases	Estimate of survival time in years
Other cases	165	5,36
PPARg high/RXR pos.	85	4,169
overall	250	4,943

Supplementary Table S6 (belonging to Figure 3C).

Subgroups	Total number of cases	Estimate of survival time in years
Other cases	136	5,369
PPARg high/RXR pos.	63	4,353
overall	199	5,04

Supplementary Table S7 (belonging to Figure 3D).

Subgroups	Total number of cases	Estimate of survival time in years
Other cases	82	5,591
PPARg high/RXR pos.	33	4,137
overall	115	5,155
